# Reviewed article: Vale D, Andrade MEC, Dantas NM. Adherence to multiple recommendations of the Dietary Guidelines for the Brazilian Population (Guia Alimentar para a População Brasileira, GAPB) and associated factors among adolescents: a cross-sectional study with data from the National Students’ Health Survey, 2019. Epidemiol Serv Saude. 2025:34;e20240404.

**DOI:** 10.1590/S2237-96222025v34e20240404.c

**Published:** 2025-06-13

**Authors:** Elisa Prezotto Giordani

**Affiliations:** FOUSP, Odontologia Social

**Table:** 

Rating	Excellent	Good	Average	Below average	Poor
Interest		✓			
Quality	✓				
Originality	✓				
Overall	✓				

**Table:** 

Questionnaire	Yes	No	Not applicable
Does the manuscript contain new and significant information to justify publication?	✓		
Does the Abstract (Summary) clearly and accurately describe the content of the article?		✓	
Is the problem significant and concisely stated?	✓		
Are the methods described comprehensively?	✓		
Are the interpretations and conclusions justified by the results?	✓		
Is adequate reference made to other work in the field?	✓		

## Manuscript Structure

Length of article is: Adequate

Number of tables is: Adequate

Number of figures is: Too short

Please state any conflict(s) of interest that you have in relation to the review of this paper (state “none” if this is not applicable): None

## Comments

Alterar o texto destacado a seguir, pois deixou a sensação de que os “fatores” estão “associados” `à adesão “muito baixa”.

“[...] recomendações do GAPB entre adolescentes é muito baixa e os fatores associados caracterizam-se por territórios [...]” 

